# Supplementary material for: Crosstalk Between Adipokines and Paraoxonase 1: A New Potential Axis Linking Oxidative Stress and Inflammation
Source: Antioxidants (Basel). 2019 Aug 6;8(8):287. doi: 10.3390/antiox8080287 (PMC6719214; doi:10.3390/antiox8080287)
Supplement: Supplementary file 1 [file antioxidants-08-00287-s001.pdf]

antioxidants

Supplementary Material

Table S1. Serum levels of adipokines and PON1 activities.

Demographic and clinical parameters	Levels
**Adipokines**	
IL-8, pg/mL	3.1 ± 2.0
TNFα, pg/mL	1.9 (1.2; 2.6)
IL-6, pg/mL	0.4 (0.3; 0.9)
Resistin, pg/mL	12.2 ± 5.2
Adiponectin, μg/mL	59 (33;118)
Leptin, ng/mL	16.1 ± 1.7
MCP-1, pg/mL	211 (159; 284)
HGF, pg/mL	265 (160; 283)
**PON1 activities**	
Arylesterase, kU/L	113 ± 29
Paraoxonase, U/L	74 (53–170)
Lactonase, U/L	104 ± 31

Data are expressed as: mean ± standard deviation for normally-distributed continuous variables; median (interquartile range) for non-normally distributed continuous variables. Abbreviations: IL (interleukin); TNF-α, tumor necrosis factor alpha; MCP-1, monocyte chemoattractant protein 1; HGF, hepatocyte growth factor.

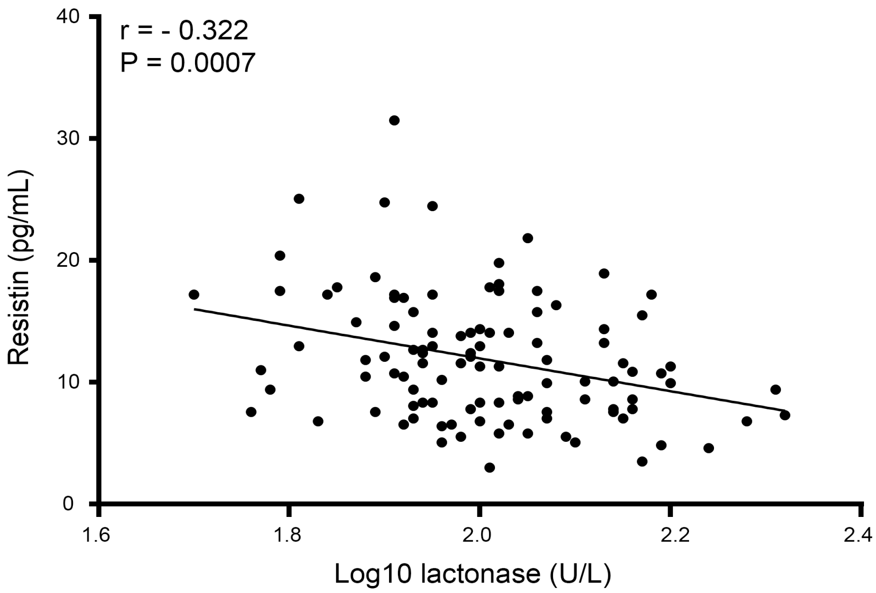

Figure S1. Inverse correlation between resistin and lactonase levels. Scatter plot showing the relationship between the circulating levels of resistin and lactonase activity levels in the whole cohort of samples. Serum levels of resistin were significantly and negatively correlated with lactonase activity.
